# Künstliche Intelligenz in der Krankenhauslogistik und in betrieblichen Prozessen

**DOI:** 10.1007/s00103-025-04094-6

**Published:** 2025-07-15

**Authors:** Sylvia Kaczmarek, Sebastian Wibbeling

**Affiliations:** Fraunhofer-Institut für Materialfluss und Logistik IML, Joseph-von-Fraunhofer-Straße 2–4, 44227 Dortmund, Deutschland

**Keywords:** Krankenhauslogistik, Künstliche Intelligenz, Betriebliche Prozesse, Prozessoptimierung, Gesundheitswesen, Hospital logistics, Artificial intelligence, Operational processes, Process optimization, Healthcare

## Abstract

Die Implementierung von KI-Lösungen in der Krankenhauslogistik gewinnt zunehmend an Bedeutung für die Optimierung krankenhausinterner Prozesse. Der vorliegende Beitrag untersucht den Einsatz künstlicher Intelligenz (KI) in der Krankenhauslogistik und weiteren betrieblichen Bereichen des Krankenhauses. Nach einer Einführung in die grundlegende Rolle der KI im Krankenhaus werden die technologischen Grundlagen von KI sowie die spezifischen Logistikprozesse eines Krankenhauses dargestellt. Eine entwickelte Matrix klassifiziert potenziell anwendbare KI-Lösungen systematisch nach ihren Automatisierungsstufen und den Organisationseinheiten eines Krankenhauses.

Anhand konkreter Anwendungsbeispiele wird das Potenzial von KI-Systemen verdeutlicht. Die KI-gestützte Transportdisposition zur Optimierung der Transportlogistik, die automatische Materialanforderung für Modulschränke im Bereich der Bestandsführung sowie die automatisierte Pflegedokumentation mittels Sensorik und maschinellen Lernens zeigen das theoretische Potenzial zur Effizienzsteigerung in betrieblichen Prozessen.

Der Beitrag identifiziert zentrale Herausforderungen bei der Implementierung von KI-Systemen im Krankenhaus, darunter technische Integration, Datenverfügbarkeit, Systemflexibilität und Transparenzanforderungen. Trotz dieser Limitationen wird deutlich, dass KI-basierte Lösungen insbesondere für die Logistik und den Betrieb von Krankenhäusern zunehmend an Bedeutung gewinnen können. Die Ergebnisse zeigen, dass der erfolgreiche Einsatz von KI-Systemen maßgeblich von der Balance zwischen technologischer Innovation und praktischer Anwendbarkeit im Krankenhausalltag abhängt. Der Beitrag fördert somit das Verständnis der Potenziale und Grenzen von KI-Anwendungen in der Krankenhauslogistik und bietet eine systematische Darstellung für deren strategische Implementierung.

## Abstract (en)

The implementation of artificial intelligence (AI) solutions in hospital logistics is becoming increasingly important for optimizing clinical processes. This paper examines the use of AI in hospital logistics and other operational areas of healthcare. Following an introduction to the fundamental role of AI in hospitals, the technological foundations and specific logistics processes are presented. A developed matrix systematically classifies the various AI solutions according to automation levels and functional areas.

The potential of AI systems is illustrated through concrete application examples. AI-supported transport disposition for optimizing transport logistics, automatic material requisition for modular cabinets in inventory management, and automated nursing documentation using sensors and machine learning demonstrate the potential for increasing efficiency in operational processes.

The analysis identifies key challenges in implementing AI systems in hospitals, including technical integration, data availability, system flexibility, and transparency requirements. Despite these limitations, it is becoming clear that AI-based solutions are gaining increasing importance, particularly for logistics and hospital operations. The results show that the successful deployment of AI systems depends significantly on the balance between technological innovation and practical applicability in everyday hospital routines. The paper thus contributes to understanding the potential and limitations of AI applications in hospital logistics and provides a systematic framework for their strategic implementation.

## Hintergrund

Das Gesundheitswesen steht vor komplexen strukturellen Herausforderungen: Der demografische Wandel führt zu einer steigenden Anzahl multimorbider Patienten, während gleichzeitig ein sich dramatisch verschärfender Fachkräftemangel die Versorgungssituation belastet. Allein in der Intensivpflege fehlten 2020 bereits 22.800 Vollzeitkräfte, während Prognosen bis 2035 sogar von 1,8 Mio. fehlenden Fachkräften im gesamten Gesundheitssektor ausgehen [1]. Die steigenden Kosten bei gleichzeitig wachsenden Qualitätsanforderungen und regulatorischen Auflagen erfordern innovative Lösungsansätze. Künstliche Intelligenz (KI) in der Logistik gilt als vielversprechende Technologie mit dem Potenzial, künftig zur Weiterentwicklung des Gesundheitssystems beizutragen [2].

Auch in Krankenhäusern hat die KI zunehmend an Bedeutung gewonnen. Die Anwendungsfelder sind sehr vielfältig. Neben den Potenzialen künstlicher Lernverfahren zur Verbesserung medizinischer Prozesse und Behandlungsergebnisse, z. B. in der bildgebenden Diagnostik, werden vermehrt KI-Verfahren zur Unterstützung der klinikinternen Logistik- und Betriebsprozesse evaluiert und erprobt. An dieser Stelle ist anzumerken, dass viele Anwendungen von KI in der Krankenhauslogistik aufgrund des speziellen Umfelds noch nicht fest etabliert sind und sich vielfach in experimentellen Phasen oder frühen Implementierungsstadien befinden. In der allgemeinen Logistikbranche hingegen sind KI-Lösungen bereits deutlich weiter verbreitet und werden erfolgreich eingesetzt.

Besonders vielversprechend erscheinen diese Potenziale angesichts der enormen logistischen Anforderungen im Krankenhaussektor. Mit durchschnittlich 15.000 unterschiedlichen Artikeln in der Materialwirtschaft und bis zu 1000 Transporten pro Tag in einem mittelgroßen Krankenhaus bietet KI ein ideales Anwendungsfeld für KI-gestützte Optimierungslösungen. Moderne Krankenhäuser sind hochkomplexe Organisationen, in denen täglich Tausende von Prozessen koordiniert werden müssen – von der Patientenaufnahme über die OP-Planung bis hin zur Versorgung und Belieferung der Funktionsbereiche mit Medikamenten und Verbrauchsmaterialien. Als zentraler Bereich gilt die Krankenhauslogistik als eines der wichtigsten Anwendungsfelder für KI im Krankenhaus. Als Querschnittsfunktion hat die Logistik Auswirkungen auf nahezu alle Bereiche des Krankenhauses und ist entscheidend für einen reibungslosen Betriebsablauf. Hier kann KI durch intelligente Automatisierung und Optimierung einen wesentlichen Beitrag zur Effizienzsteigerung leisten.

Die Optimierung logistischer Prozesse durch KI kann nicht nur Kosten senken, sondern auch die Versorgungsqualität verbessern und das Personal entlasten [3]. Die Integration von KI-Systemen in bestehende Krankenhausstrukturen erfordert dabei eine enge Abstimmung zwischen IT-Infrastruktur, Krankenhausinformationssystem (KIS) und den spezifischen Anforderungen der einzelnen Fachabteilungen.

Der vorliegende Beitrag gibt einen Überblick über die Potenziale und Anwendungsmöglichkeiten von KI im Krankenhaus – insbesondere in der Krankenhauslogistik. Nach einer kurzen Darstellung der technologischen Grundlagen werden Einsatzmöglichkeiten von KI in patienten- und materialbezogenen Logistik- und Transportprozessen erläutert. Im Anschluss werden drei aktuelle Anwendungsbeispiele vorgestellt. Abschließend erfolgt eine Diskussion der Voraussetzungen und Potenziale von KI im Krankenhaus sowie ein Ausblick auf zukünftige Entwicklungen.

## Technologische Grundlagen und Funktionsweise der KI in der Krankenhauslogistik

Eingesetzte Technologien in der Krankenhauslogistik umfassen derzeit überwiegend klassische, regelbasierte Ansätze – oft auch als „symbolische KI“ bezeichnet. Während viele aktuell eingesetzte Systeme noch auf diesen festen Entscheidungsregeln beruhen, gewinnen datenbasierte KI-Verfahren, insbesondere das maschinelle Lernen, welches sich methodisch in überwachtes, unüberwachtes und teilüberwachtes Lernen unterteilt [4], zunehmend an Bedeutung.

Als Teilgebiet der Informatik zielt KI darauf ab, Systeme zu entwickeln, die menschenähnliche kognitive Funktionen wie Lernen, Problemlösen und Sprachverständnis nachbilden können. Seit den Anfängen der KI-Forschung in den 1950er-Jahren, markiert durch Turings wegweisenden Test zur Unterscheidung zwischen Menschen und Maschinen [5], hat sich insbesondere das maschinelle Lernen als zentrales Forschungsfeld etabliert. In der Praxis entstehen dabei verschiedene Formen der Mensch-Maschine-Zusammenarbeit, die durch unterschiedliche Automatisierungsgrade gekennzeichnet sind – von vollständig manuellen bis hin zu komplett automatisierten Ausführungen [6].

**Abb. 1:**
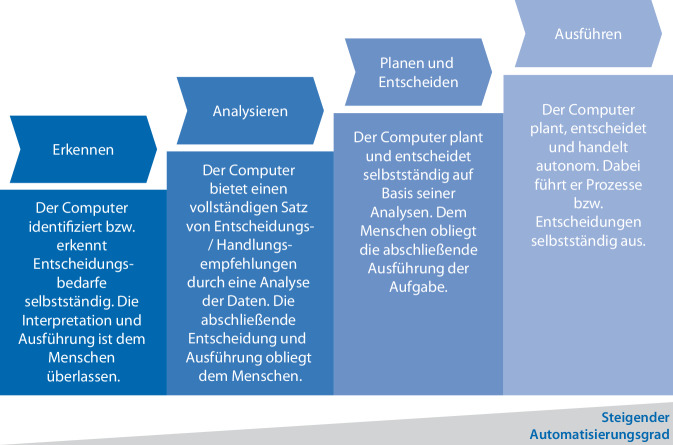
Stufen der Automatisierung und kognitiven Fähigkeiten in der Zusammenarbeit zwischen Menschen und künstlicher Intelligenz [7]

Es lassen sich vier Automatisierungsstufen unterscheiden (Abb. 1): Die *erste Stufe* der Automatisierung umfasst das Erkennen und Identifizieren. Der Computer erkennt eigenständig Entscheidungsbedarfe und leitet diese an den Nutzer weiter. Die Interpretation der Daten sowie die daraus abgeleiteten Handlungen verbleiben vollständig beim Menschen. In der *zweiten Stufe*, dem Analysieren, geht das System einen Schritt weiter und generiert durch eine umfassende Datenanalyse konkrete Handlungsempfehlungen. Auch hier verbleiben die finale Entscheidungsgewalt und die Umsetzung beim Menschen, der die vorgeschlagenen Optionen prüft und auswählt. Die *dritte Stufe*, Planen und Entscheiden, überträgt dem Computer bereits weitreichendere Kompetenzen. Das System analysiert nicht nur die Situation, sondern entwickelt eigenständig Pläne und trifft auf deren Basis auch die entsprechenden Entscheidungen. Der Mensch behält hier lediglich die Kontrolle über die tatsächliche Durchführung der beschlossenen Maßnahmen. In der *vierten* und höchsten *Automatisierungsstufe*, dem Ausführen, agiert der Computer vollständig autonom. Er vereint alle vorherigen Kompetenzen und führt die Prozesse eigenständig aus, sodass der gesamte Vorgang von der Planung bis zur Umsetzung ohne menschliches Eingreifen erfolgt. Im Folgenden werden die Anwendungsfälle von KI in der Krankenhauslogistik entlang der Automatisierungsstufen systematisiert.

Im Vergleich zur medizinischen Diagnostik, die sich hauptsächlich auf Entscheidungsunterstützungssysteme zur Detektion und Prädiktion und damit auf KI-Systeme der ersten und zweiten Stufe stützt, eröffnet die *Krankenhauslogistik* weiterreichende Möglichkeiten zur Prozessautomatisierung mittels KI über die zweite Automatisierungsstufe hinaus. Ein anschauliches Beispiel dafür sind autonome mobile Roboter (AMR). Sie werden der vierten Stufe zugeordnet. Mithilfe von Schwarmintelligenz in Multiagentensystemen, die auf verstärkendem Lernen basieren, passen sie ihre Routenplanung selbstständig und dynamisch an verschiedene Störfaktoren wie physische Hindernisse oder Prozessänderungen an und führen Transporte eigenständig durch.

Obwohl viele datengetriebene KI-Systeme in der Krankenhauslogistik aktuell noch nicht flächendeckend eingesetzt werden, sind sie in der Logistikwelt bereits vielfach erprobt und etabliert – und könnten künftig auch im Gesundheitswesen eine stärkere Rolle spielen. Um diese Potenziale der KI in der Krankenhauslogistik besser zu verstehen, werden im folgenden Abschnitt die unterschiedlichen Logistikprozesse eines Krankenhauses detailliert betrachtet.

## Die Logistikprozesse eines Krankenhauses

Im Krankenhaus bilden verschiedene Funktionsbereiche ein komplexes und ineinandergreifendes System, das eine umfassende und qualitativ hochwertige Patientenversorgung ermöglicht. Ein wichtiger Bestandteil dieses Systems sind die dezentralen *Logistikbereiche des Krankenhauses* und der klassische *Transport*. Hier wird durch patienten- und materialbezogene Logistik- und Transportprozesse sichergestellt, dass sowohl die Bereitstellung von Materialien als auch Patiententransfers zu Stationen oder Behandlungen effizient und zielgerichtet erfolgen. Zu den zentralen logistischen Aufgaben zählen beispielsweise die Speisenversorgung sowie die Betten- und Wäschelogistik (Transport, Aufbereitung).

Das *Zentrallager* gewährleistet die kontinuierliche Verfügbarkeit von medizinischen und nichtmedizinischen Verbrauchsmaterialien. Die *Apotheke* stellt nicht nur die Versorgung mit Medikamenten sicher, sondern berät auch das medizinische Personal zur sicheren Anwendung, was besonders bei komplexen Behandlungsplänen wichtig ist.

Ein zentraler Bereich der Krankenhausorganisation ist die operative und pflegerische Versorgung. Der *OP-Bereich* ist mit modernster Technik ausgestattet und stellt durch standardisierte und spezialisierte Abläufe die Durchführung präziser medizinischer Eingriffe sicher. Nach erfolgten operativen Eingriffen oder bei allgemein stationärer Aufnahme übernehmen die *Pflegestationen* die kontinuierliche medizinische und pflegerische Betreuung der Patienten. Die Pflegekräfte gewährleisten durch eine ganzheitliche Pflege eine patientenzentrierte Versorgung.

Für den OP-Bereich sowie zur Einhaltung und Sicherstellung von Hygienestandards ist die *Aufbereitungseinheit* *für* *Medizinprodukte* *(AEMP)* unerlässlich. Sie sorgt nicht nur für die sachgerechte Reinigung, Desinfektion und Sterilisation von medizinischen Instrumenten, sondern spielt auch eine zentrale Rolle bei der effizienten Bereitstellung dieser Instrumente. Zudem unterstützt die AEMP nicht nur die OP-Bereiche, sondern auch andere interventionelle Funktionseinheiten, was ihre Bedeutung innerhalb des gesamten Krankenhausbetriebs unterstreicht.

Die *Notaufnahme* fungiert als primäre Anlaufstelle für akute medizinische Notfälle, wo durch rasche Diagnostik eine sofortige Behandlungsentscheidung getroffen werden muss und in der Regel bereits ambulant behandelt werden kann. Für die Behandlung von Patienten, die keiner stationären Aufnahme bedürfen, stehen die Ambulanzen und Polikliniken zur Verfügung. Dadurch wird die stationäre Infrastruktur entlastet und eine kontinuierliche Versorgung auf ambulanter Ebene sichergestellt.

**Abb. 2:**
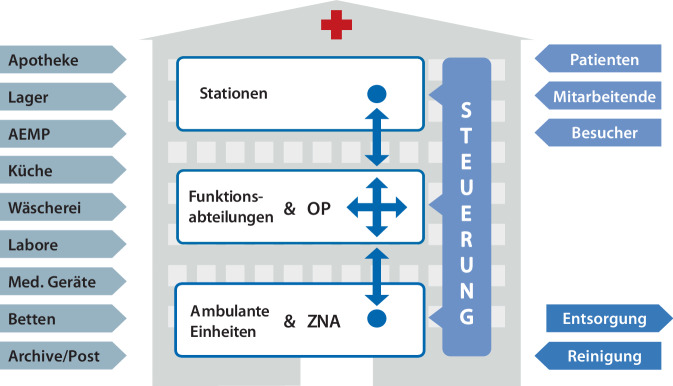
Die logistischen Ströme eines Krankenhauses (in Anlehnung an [8]). *AEMP* Aufbereitungseinheit für Medizinprodukte, *OP* Operationsbereich, *ZNA* zentrale Notaufnahme

Abb. 2 fasst die logistischen Ströme eines Krankenhauses zusammen. Ausgehend von dieser Struktur der Organisationseinheiten lassen sich konkrete Einsatzmöglichkeiten für KI-Anwendungen ableiten. Um eine strukturierte Übersicht dieser Anwendungspotenziale zu erhalten, werden im folgenden Abschnitt KI-Systeme für die spezifischen Organisationseinheiten dargestellt.

## Anwendung von KI zur Unterstützung in der Krankenhauslogistik

Für die wissenschaftliche Erforschung des KI-Einsatzes ist ein systematischer Überblick über vorhandene Anwendungen und Zukunftsaussichten essenziell. Tab. 1 setzt die zuvor beschriebenen Automatisierungsstufen (Abb. 1) in Beziehung zu den spezifischen Organisationseinheiten des Krankenhauses (Abb. 2). Diese Matrixdarstellung bietet eine strukturierte Übersicht über potenziell anwendbare KI-Lösungen im Krankenhaus, wobei der Fokus auf Technologien liegt, die sich in der Logistikwelt bereits etabliert haben. Es ist wichtig zu betonen, dass sich die aufgeführten Lösungen für Krankenhäuser überwiegend noch in der Entwicklungs- oder Forschungsphase befinden und bislang nur in Einzelfällen marktfähig oder in frühen Implementierungsstadien sind. Tab. 1 verdeutlicht somit die vielversprechenden Einsatzmöglichkeiten dieser Technologien in verschiedenen Krankenhausbereichen und lässt sich flexibel um neue Entwicklungen erweitern.

**Tab. 1:** KI-Anwendungen in der Krankenhauslogistik nach Automatisierungsstufen und Organisationseinheiten [7]

Automatisierungsstufen	Organisationseinheiten				
	Transport/Logistik	Zentrallager/Apotheke	OP-Bereich	Pflegestation	AEMP
*1) Erkennen*	Tracking von Objekten/Personal/ Geräten	–	Tracking von med. Geräten		Qualitätsprüfung
	Verfügbarkeit von Ressourcen	Intelligentes Lager/intelligenter Warenein-/-ausgang	Verbrauchsdokumentation	Patientenbewegungen	
	Identifikation von Objekten/Transportwagen/Versorgungsrisiken/Raumbedingungen (Hygiene/Saalzustand)				
	–	–	Sprach‑, Gesten- oder Gesichtserkennungen		
			Erkennen und Sammeln von Echtzeitdaten/Materialverbrauch		
			Klassifizierung von Operationen	–	Zustand der Instrumente/Erkennung von Schmutzresten
*2) Analysieren*	Analyse von Bewegungsdaten zur Ermittlung der Transportbedarfe	Intelligente Kommissionierung/Lagerverwaltung	OP-Verlauf/Performanceanalysen	Analyse des Behandlungsfortschritts auf Basis von Materialerkennung	Analyse der Siebzusammenstellung/OP-spezifische Verbräuche
	–	–	Intelligente Belegungsplanung durch Analyse der Saalauslastung/AWR	Bedarfs‑/Verbrauchsanalysen	Erkennung und Analyse zur Instandhaltung und Wartung der Anlagen
*3) Planen und Entscheiden*	Intelligente Transportdisposition/Aufzugssteuerung	Materialbedarfsprognosen, Bestandsmengenoptimierung, Retourenmanagement			
	Problembehebung bei der Fahrt	–	Personalplanung mit gesetzlichen Regelungen (Qualifikationen) als Reaktion auf kurzfristige Ausfälle		
	Intelligentes Packen		Erstellung von OP-Plänen und Saalbelegung auf Basis von prognostizierten OP-Dauern	Bettenbedarfsplanung/-prognosen	Siebpriorisierung
*4) Ausführen*	Autonome Transporte	Autonome Roboter			
		Intelligentes Warenmanagement (z. B. automatische Bestellungen)			
		Steuerung der Lagertechnik, Kommissionierung und Warenausgang	Assistenzsysteme (z. B. Richtprozess)	Ausführung patientenferner Tätigkeiten (sprach-/bildbasiert), z. B. Dokumentation	Automatisches Fallwagenlager mit intelligenter Lagerplatzvergabe und -verwaltung und Kommissionierung

*AEMP* Aufbereitungseinheit für Medizinprodukte, *AWR* Aufwachraum, *OP* Operation

### KI-Anwendungen in Logistik/Transport

Die Logistik stellt einen fundamentalen Bestandteil eines funktionsfähigen Krankenhauses dar. Die aktuellen fortschrittlichen Möglichkeiten von Sensorik, beispielsweise zur Identifikation, Lokalisierung oder Analyse von Bewegungs‑, Last- und Betriebsdaten verschiedener technischer Systeme wie Transport- und Handlingsroboter, eröffnen vielfältige Anwendungsszenarien für künstliche Intelligenz [7].

Im klassischen Transport ermöglichen autonome Transportroboter mit dezentralen Steuerungsmechanismen einen intelligenten Ressourceneinsatz durch schwarmintelligente Verhaltensweisen und optimierte Auftragsdisposition. Schwarmintelligente Verhaltensweisen beziehen sich auf die Fähigkeit dieser Systeme, durch Zusammenarbeit und Kommunikation zwischen mehreren Robotern optimale Entscheidungen zu treffen, ähnlich, wie es in der Natur beobachtet wird. Diese Systeme nutzen Algorithmen des maschinellen Lernens, um Muster in den Daten zu erkennen und Entscheidungen in Echtzeit zu treffen, was die Effizienz und Geschwindigkeit des Transports erheblich steigert (intelligente Wegeführung, Vermeidung von Leerfahrten, Nutzung von verfügbaren Aufzügen etc.; [7]). Einen Schritt weiter gehen Transportroboter mit KI-fähigen Sensorboxen, welche sich derzeit in der Entwicklungsphase befinden und den Grundgedanken innovativer Logistikansätze aufgreifen. Mit ihnen können autonome Fahrzeuge aus menschlichen Interventionen bei Transportstörungen lernen und zukünftige ähnliche Situationen selbstständig bewältigen. Dies reduziert nicht nur die Notwendigkeit menschlicher Eingriffe, sondern optimiert auch den Gesamtprozess [9]. Mit KI können sowohl prädiktive Planungsansätze unter Berücksichtigung dynamischer Einflussfaktoren als auch flexible Reaktionsmöglichkeiten auf unerwartete Ereignisse umgesetzt werden [7].

### KI-Anwendungen im Zentrallager/Apotheke

Auch im Kontext von Zentrallagern und Krankenhausapotheken bietet künstliche Intelligenz vielversprechende Anwendungspotenziale. Im Rahmen der Versorgung von Materialien lassen sich durch Predictive Analytics bereits in anderen Branchen präzise Versorgungs- und Beschaffungsrisiken identifizieren. Die KI generiert Bedarfsprognosen für erforderliche Artikelmengen, wodurch nicht nur optimale Lagermengen vorhergesagt, sondern auch die Lagerverwaltung unterstützt wird. Hierbei erfolgt eine intelligente Analyse unterschiedlicher Faktoren, die z. B. physische Produkteigenschaften und Lagerzonen umfasst [7].

In der Lagerlogistik kann KI somit prädiktive Planungsansätze ermöglichen, die dynamische Einflussfaktoren berücksichtigen. Darüber hinaus bieten sie flexible Reaktionsmöglichkeiten auf unerwartete Ereignisse. Beispielsweise können KI-gestützte Systeme historische Daten analysieren, um zukünftige Nachfragen vorherzusagen und Lagerbestände anzupassen, wodurch die Verfügbarkeit der Ressourcen verbessert wird [7].

In den Kommissionierprozessen kann KI die wahrscheinlichen Mengen pro Kostenstelle vorhersagen, was den Einsatz von Transportmitteln effizienter gestaltet. Mit der Weiterentwicklung der Technologie wird erwartet, dass KI-gestützte Kommissionier- und Transporttechnik, insbesondere autonome Robotersysteme, zunehmend etabliert werden. Diese Systeme optimieren die Materialflussprozesse innerhalb verschiedener Organisationseinheiten und tragen zur Steigerung der Gesamtproduktivität bei [7].

### KI-Anwendungen im OP-Bereich

Künstliche Intelligenz bietet im OP-Bereich, einem wirtschaftlich kritischen Krankenhausbereich, vielfältige Anwendungsmöglichkeiten, die potenziell nicht nur die Effizienz steigern, sondern auch die Patientensicherheit erhöhen. Obwohl sich viele dieser Technologien noch in der Entwicklungsphase befinden, zeigen Fortschritte in der Sprach‑, Gesten- und Gesichtserkennung, dass sie zukünftig zur Identifikation und Überwachung von Operationen und Arbeitsabläufen eingesetzt werden könnten. Solche Systeme könnten die Kommunikation im OP-Team verbessern und die Entscheidungsfindung in Echtzeit unterstützen – und so die Patientensicherheit erhöhen. Ein weiteres wichtiges Einsatzszenario könnte die KI-gestützte Bewertung des hygienischen Zustands eines OP-Saals sein. Durch die Analyse von Bilddaten und Sensormessungen könnte KI die Sterilität überwachen und notwendige Maßnahmen zur Minimierung von Infektionsrisiken ableiten, was entscheidend für die Patientensicherheit ist [7].

Im materialintensiven OP-Bereich könnten KI-Technologien künftig präzise Bedarfs- und Verbrauchsprognosen durchführen, was die Materialbeschaffung optimieren und die automatisierte Dokumentation des Materialverbrauchs für das DRG-Controlling (Diagnosis-related Groups) unterstützen könnte, wodurch die Abrechnung effizienter gestaltet werden kann [7].

Big-Data-Verfahren können außerdem die Dauer von Operationen sowie die Belegung von OP-Sälen prognostizieren, wodurch die Planung und Ressourcennutzung (Material und Personal) verbessert werden. Zukünftig wird auch der Einsatz KI-gestützter Robotik erwartet, um Materialfluss- und Instrumentenprozesse zu unterstützen und das Personal von zeitaufwendigen Aufgaben zu entlasten [7].

### KI-Anwendungen in einer Pflegestation

In bettenführenden Bereichen wie Pflege- und Intensivstationen eröffnet KI innovative Möglichkeiten zur Optimierung des Materialmanagements und der Logistikprozesse. Eine KI-unterstützte Analyse von Materialverbrauchsdaten ermöglicht präzise Vorhersagen für optimale Lager- und Bestellmengen. Durch automatisierte, sensorgestützte Erkennungen von Materialentnahmen und leeren Materialfächern lassen sich beispielsweise Materialbedarfe erkennen und Anforderungsprozesse automatisch auslösen. Dies gewährleistet eine kontinuierliche Verfügbarkeit von notwendigen Materialien, was in kritischen Pflegeumgebungen von großer Bedeutung ist [10].

Darüber hinaus bietet KI das Potenzial, Pflegetätigkeiten durch Bewegungsidentifikation mittels Sensorik automatisch zu erkennen und zu dokumentieren. Dies verbessert nicht nur die Nachverfolgbarkeit von Pflegeleistungen, sondern entlastet das Pflegepersonal von Dokumentationsaufgaben [11].

In Bereichen der Robotik können Logistikprozesse in Pflegestationen durch autonom agierende Robotersysteme unterstützt werden. Diese Systeme können Materialien, Medikamente und Laborproben effizient zwischen verschiedenen Krankenhausbereichen, beispielsweise zwischen Intensivstation und Labor, transportieren [7].

### KI-Anwendungen in einer Aufbereitungseinheit für Medizinprodukte (AEMP)

Die Aufbereitungseinheit für Medizinprodukte (AEMP) stellt einen kritischen Funktionsbereich der Krankenhauslogistik dar, der hohe manuelle Ressourcen für Qualitätsansprüche und zuverlässige Versorgung erfordert. Auch hier bieten KI-basierte Systeme innovative Lösungsansätze zur Prozessoptimierung. [7].

Ein Beispiel ist die komplexe Analyse der Medizinproduktaufbereitung. Durch Erfassung von Zustandsinformationen, z. B. über kamerabasierte Systeme, können Instrumentenverbräuche mit maschinellen Lernsystemen ausgewertet und fall- sowie arztspezifische Merkmale intelligent berücksichtigt werden. So wird eine präzise Steuerung der Instrumentenlogistik, beginnend mit der automatisierten Bestandserfassung bis hin zur vorausschauenden Bedarfsplanung, ermöglicht. Dies fördert eine optimale Versorgung der OPs mit notwendigen Instrumenten und reduziert gleichzeitig das Risiko von Engpässen [7].

Eine vollautomatisierte Planung der Verfügbarkeit chirurgischer Instrumentensiebe kann den Nutzungsgrad von Instrumenten steigern und gleichzeitig die Lagerbestände signifikant reduzieren. Besonders in Kombination mit Fallwagensystemen ergeben sich Vorteile durch Flächeneinsparungen und optimierte Prozesse. Auf dem Weg von der AEMP in den OP können mittels sensorischer Erfassung Transportmittel, Siebe und Instrumente intelligent koordiniert werden. Insgesamt können KI-unterstützte Planungen so zu einem höheren Nutzungsgrad einzelner Instrumente bei gleichzeitiger Bestandsreduzierung führen [7].

## Aktuelle Anwendungsbeispiele für KI in der Krankenhauslogistik

### Transportlogistik – KI-gestützte Transportdisposition im Krankenhaus

Die interne Transportlogistik ist ein entscheidender Erfolgsfaktor im Krankenhaus, da sie direkt die Effizienz der Behandlungsabläufe beeinflusst. Herkömmliche Dispositionssysteme arbeiten derzeit mit fest programmierten Algorithmen zur Verteilung von Transportaufträgen. Diese starren Systeme stoßen jedoch schnell an ihre Grenzen, besonders wenn sich die Verfügbarkeit von Personal und Ressourcen kurzfristig ändert oder unerwartete Situationen eintreten [12]. Die wachsende Komplexität der Krankenhauslogistik und die Notwendigkeit, schnelle Entscheidungen zu treffen, überfordern zunehmend diese klassischen Optimierungsansätze.

**Abb. 3:**
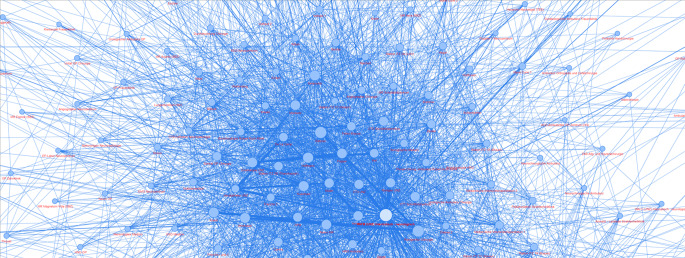
Netzwerk zur Visualisierung von Transportbeziehungen zwischen Funktionsbereichen eines Krankenhauses zur Analyse des optimalen Transportwegs [12]

Ein vom ehemaligen Bundesministerium für Bildung und Forschung (BMBF) gefördertes Forschungsvorhaben „Künstliche Intelligenz zur Prognose und Steuerung in der Krankenhaus-Transportdisposition – KIK_Dispo“ untersuchte beispielsweise das Potenzial innovativer KI-Lösungen in diesem Bereich. Abb. 3 zeigt eine Netzwerkvisualisierung, die typischerweise bei der Analyse von Transportbeziehungen verwendet wird. Die Knoten im Bild repräsentieren die verschiedenen Organisationseinheiten. Die Linien (Verbindungen) zwischen diesen Knoten zeigen die Transportbeziehungen. KI wird verwendet, um Muster oder Anomalien in diesen Netzwerken zu erkennen, was dabei helfen kann, effizientere Transportwege zu planen oder Engpässe zu identifizieren. Dabei zeigte sich, dass die KI-gestützte Transportdisposition durch maschinelles Lernen eine intelligente automatische Steuerung ermöglicht, die aus Erfahrungen lernt und sich kontinuierlich verbessert. Das System berücksichtigt dabei verschiedene Faktoren gleichzeitig – von typischen Auslastungszeiten über verfügbares Personal bis hin zur Dringlichkeit verschiedener Transporte [13].

Die praktischen Ergebnisse solcher KI-Anwendungen sind überzeugend: Studien belegen eine verbesserte Effizienz von fahrerlosen Transportfahrzeugen von 20–30 % [14]. Besonders wichtig ist dabei die Entlastung des Pflegepersonals, welches beispielsweise durch entfallende Botengänge mehr Zeit für seine eigentlichen pflegerischen Aufgaben gewinnt. Dies führt nachweislich zu einer besseren Versorgungsqualität und hilft, die Herausforderungen des Fachkräftemangels besser zu bewältigen.

### Bestandsführung – KI-gestützte automatische Materialanforderung für Modulschränke

Die effiziente Bestandsführung von Medizin- und Verbrauchsmaterial auf Krankenhausstationen stellt eine zentrale logistische Herausforderung dar. Insbesondere in Modulschranksystemen, die nach dem „Kanban-Prinzip“ betrieben werden, ist eine präzise und zeitnahe Nachbestellung essenziell für die Versorgungssicherheit [15]. Traditionell basiert dieses System auf manuellen Prozessen, bei denen Pflegekräfte oder Versorgungsassistenten die Materialbestände überwachen und Nachbestellungen über Barcodesysteme auslösen müssen.

Die manuelle Bestandsführung birgt jedoch erhebliche Risiken: Im hektischen Stationsalltag kommt es häufig zu Fehlern bei der Kennzeichnung leerer Fächer oder vergessenen Nachbestellungen. Dies führt einerseits zu kritischen Materialengpässen, andererseits zu überhöhten Beständen durch Doppelbestellungen [15]. Zudem bindet diese logistische Tätigkeit wertvolle Zeit des pflegerischen Personals.

**Abb. 4:**
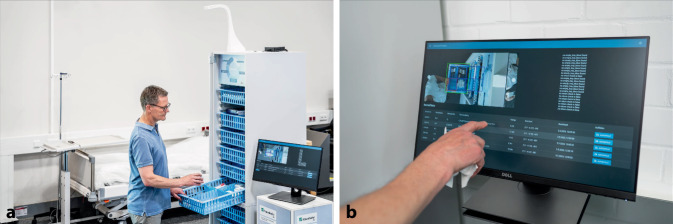
Automatische Bestellanforderung mittels Objekterkennung und KI-System „AI4Demand“. **a** Erfassung der einzelnen Modulkörbe mittels Kamera; **b** Bildauswertung mittels KI, automatische Nachbestellung der leeren Fächer. (*Quelle*: Fraunhofer IML)

Ein innovativer Lösungsansatz, der im Rahmen eines Forschungsprojekts „AI4Demand“ des Fraunhofer-Institutes für Materialfluss und Logistik im Rahmen der Silicon Economy entwickelt wurde, nutzt KI-gestützte Bildverarbeitung zur Automatisierung dieser Prozesse (Abb. 4a, b). Das System basiert auf intelligenten Kameraeinheiten, die über den Modulschränken installiert werden und mittels Bewegungserkennung aktiviert werden. Sobald ein Modulkorb zur Materialentnahme geöffnet wird, analysiert eine trainierte KI in Echtzeit den Füllstand der einzelnen Fächer. Durch umfangreiches Training mit Methoden des maschinellen Lernens erkennt die KI zuverlässig leere Fächer, auch unter verschiedenen Umgebungsbedingungen und bei teilweiser Verdeckung. Die automatische Bestellauslösung erfolgt direkt über eine Anbindung an das Krankenhausbestellsystem, wobei die gesamte Bildverarbeitung aus Datenschutzgründen lokal auf den Kamerageräten erfolgt [15].

Die praktischen Vorteile dieser KI-Anwendung sind signifikant, denn sie führt zu einer hohen Zeitersparnis für das Versorgungspersonal und zu einer optimierten Materialverfügbarkeit [15]. Dies führt nicht nur zu einer verbesserten Flächenauslastung durch Bestandsreduktion, sondern ermöglicht es dem Pflegepersonal, sich stärker auf seine Kernaufgaben in der Patientenversorgung zu konzentrieren.

### Pflegedokumentation – KI-gestützte Automatisierung durch Sensorik und Deep Learning

Die Dokumentation von Pflegetätigkeiten ist ein zentraler, jedoch zeitaufwendiger Bestandteil des Klinikalltags. Pflegekräfte verbringen täglich durchschnittlich 52 min je Schicht damit, erbrachte Leistungen manuell zu erfassen, um Abrechnungsgrundlagen zu schaffen und Qualitätsstandards einzuhalten [16]. Diese Tätigkeit bindet wertvolle Ressourcen, ohne direkt zur Patientenversorgung beizutragen.

Ein innovativer Ansatz zur Entlastung des Pflegepersonals basiert auf einer KI-gestützten Lösung, die durch Sensorik und Machine Learning unterstützt wird. Im Rahmen eines Forschungsprojektes „Eingabefreie Station“, gefördert durch das Rahmenprogramm „EFRE.NRW“ (Europäischer Fonds für regionale Entwicklung/Land Nordrhein-Westfalen) im Rahmen des Leitmarktwettbewerbs „Gesundheit.NRW“, wurde eine Technologie entwickelt, bei der mobile Sensoren und Funksender Pflegetätigkeiten automatisch erfassen und in Echtzeit analysieren. Mithilfe von maschinellem Lernen werden die Tätigkeiten präzise den entsprechenden Tätigkeitsnachweisen zugeordnet. Eine nachträgliche Prüfung und Bestätigung durch das Pflegepersonal erfolgt über ein mobiles Endgerät und überträgt die geleisteten Pflegetätigkeiten abschließend in die Patientenakte. Durch dieses System werden Dokumentationsprozesse effizienter gestaltet, indem der Zeitaufwand reduziert wird und gleichzeitig Fehlerquellen minimiert werden. Durch die lokale Verarbeitung der sensiblen Daten auf den Endgeräten wird den hohen Anforderungen an Datenschutz und Anonymisierung Rechnung getragen [11]. Die automatisierte Dokumentation ermöglicht eine spürbare Entlastung der Pflegekräfte, wodurch mehr Zeit für die direkte Interaktion mit Patienten bleibt.

## Diskussion des Potenzials und Ausblick

Das Potenzial von KI-Lösungen in der Krankenhauslogistik zeigt sich insbesondere in ihrer vielseitigen Anwendbarkeit in den unterschiedlichen Bereichen und Organisationseinheiten. Die vorgestellten Anwendungsbeispiele verdeutlichen, dass KI-Systeme in verschiedenen Funktionsbereichen des Krankenhauses spezifische Verbesserungspotenziale bieten können. Dies erlaubt eine differenzierte Betrachtung ihrer Bedeutung und Wirksamkeit in den verschiedenen Einsatzgebieten und hebt hervor, dass in verschiedenen betrieblichen Prozessen eines Krankenhauses gezielte Anwendungsmöglichkeiten für KI existieren.

Die systematische Einordnung der KI-Lösungen anhand von Automatisierungsstufen und Organisationseinheiten, wie sie in Tab. 1 dargestellt wurde, verdeutlicht dabei die enorme Bandbreite der Anwendungsmöglichkeiten. Diese strukturierte Betrachtung ermöglicht es Krankenhäusern, den Einsatz von KI strategisch zu planen und dabei sowohl die technologische Reife als auch die spezifischen Anforderungen der verschiedenen Funktionsbereiche zu berücksichtigen. Die entwickelte Matrix bietet damit nicht nur einen Überblick über den Status quo, sondern auch eine flexible Grundlage für die Integration zukünftiger Entwicklungen.

Um dieses Potenzial in der Praxis erfolgreich zu erschließen, gilt es jedoch, eine Reihe spezifischer Herausforderungen zu bewältigen, die sich bei der Implementierung und Nutzung von KI-Systemen im Krankenhausumfeld ergeben:

### *Technische Voraussetzungen.*

Bei der Implementierung von KI in der Krankenhauslogistik sind spezifische Herausforderungen zu bewältigen. Eine zentrale technische Hürde ist die Sicherstellung einer stabilen technischen Infrastruktur, einschließlich WLAN-Ausstattung. Erfolgreiche Implementierungen zeigen jedoch, dass sich die Investition in KI-gestützte Logistiklösungen sowohl wirtschaftlich als auch qualitativ auszahlt [17].

### *Datenqualität und -verfügbarkeit.*

Eine weitere kritische Voraussetzung für den Erfolg von KI-Anwendungen ist der Zugang zu hochwertigen, umfangreichen und gut strukturierten Daten. Die im Gesundheitswesen häufig anzutreffende Fragmentierung von Daten sowie deren unvollständige oder erschwerte Zugänglichkeit stellen bedeutende Hindernisse dar. Insbesondere in nichtmedizinischen Bereichen stehen in Krankenhäusern oftmals keine hochwertigen Daten zur Verfügung, sodass ergänzende Ansätze erforderlich sind – etwa Human-in-the-Loop-Konzepte (HITL), bei denen Experten während der Nutzung von KI-Systemen einbezogen werden. Durch diese Kombination von künstlichem und menschlichem Expertenwissen entstehen leistungsstärkere und kosteneffiziente KI-Anwendungen [18]. Zudem erfordert die erfolgreiche Implementation eine Standardisierung der Datenformate und die Gewährleistung der Interoperabilität zwischen verschiedenen IT-Systemen.

Von Bedeutung ist zudem die Schaffung von *Transparenz und Vertrauen.* Gerade im sensiblen Bereich des Gesundheitswesens birgt eine als „Blackbox“ wahrgenommene KI das Risiko mangelnder Akzeptanz. Es gilt, ein ausgewogenes Maß an Nachvollziehbarkeit zu etablieren, das Vertrauen in die Entscheidungsfindung schafft, ohne dabei die Nutzer zu überfordern [19].

Abschließend lässt sich festhalten, dass die Integration von KI in die Krankenhauslogistik trotz der genannten Herausforderungen unaufhaltsam voranschreitet. Die Technologie wird dabei zunehmend ausgereifter und anwenderfreundlicher. Künftige Entwicklungen werden vor allem auf eine verbesserte Integration der Systeme, erhöhte Adaptivität und gesteigerte Transparenz abzielen. Der Schlüssel zum Erfolg liegt in der Balance zwischen technologischer Innovation und praktischer Anwendbarkeit im komplexen Krankenhausalltag.

Für den *Einstieg in die KI-Transformation* der Krankenhauslogistik bieten sich insbesondere KI-gestützte Bildverarbeitungs- und Robotiklösungen an. Robotiktechnologien sind in der allgemeinen Logistik und Produktion bereits fest etabliert, marktreif und vielfach in der Anwendung. Diese Technologien verfügen somit bereits über ausgereifte Frameworks aus anderen Wirtschaftszweigen, die sich einfacher auf die Krankenhauslogistik übertragen lassen. Wesentliche *betriebliche Potenziale* können somit durch die Integration solcher Systeme erreicht werden. Die Realisierungshürden sind niedriger, da auf bewährte Lösungen aus Industrie und anderen Sektoren zurückgegriffen werden kann. Vor allem Transportaufgaben und Prozessautomatisierungen lassen sich mit geringem Entwicklungsaufwand adaptieren [20]. Diese Technologien ermöglichen einen risikoarmen und schrittweisen Einstieg in die digitale Transformation und bieten Krankenhäusern die Chance, erste praktische Erfahrungen mit KI-Systemen zu sammeln.
